# How to mathematically optimize drug regimens using optimal control

**DOI:** 10.1007/s10928-018-9568-y

**Published:** 2018-02-06

**Authors:** Helen Moore

**Affiliations:** 1 Bristol-Myers Squibb, Route 206 & Province Line Road, Princeton, NJ 08543 USA; 2 Present Address: AstraZeneca, 35 Gatehouse Drive, Waltham, MA 02451 USA

**Keywords:** Optimal control, Constrained optimization, Combination therapy, Disease modeling, Control theory

## Abstract

This article gives an overview of a technique called *optimal control*, which is used to optimize real-world quantities represented by mathematical models. I include background information about the historical development of the technique and applications in a variety of fields. The main focus here is the application to diseases and therapies, particularly the optimization of combination therapies, and I highlight several such examples. I also describe the basic theory of optimal control, and illustrate each of the steps with an example that optimizes the doses in a combination regimen for leukemia. References are provided for more complex cases. The article is aimed at modelers working in drug development, who have not used optimal control previously. My goal is to make this technique more accessible in the biopharma community.

## Introduction

One month before our June 2011 wedding, my husband Colin easily completed a 100-mile bike ride (and bragged that he passed many of the other cyclists, but none passed him). One month after our wedding, he was diagnosed with stage 4 metastatic gastric cancer. Without much warning, we were faced with an urgent question: which treatment option to choose? Admittedly, the options were limited and not very effective for gastric cancer.

In a situation with limited time and resources, how do we determine the best possible treatment for a patient with a given disease? That is, without the luxury of more than a decade or two billion dollars [1], what is the best we can do with the currently-available therapies?

Optimal control provides one potential way to answer this question, and it requires much less time and money than it takes to develop a new drug. It requires close collaboration among team members with disease knowledge and computational expertise, and access to a differential equation solver. If we create a semi-mechanistic model of the disease, we can optimize combination regimens in silico for the drugs currently available. This is particularly useful when we have so many potential therapies for a disease, that there are too many combinations of dose levels to test, even preclinically (in animal studies). This technique can also be used to optimize doses and schedules for new compounds, especially when they are being tested in combination with other therapies. If our model is good enough, then, by design, the optimal control regimen should be at least as good as any other, but could be much better.

The optimal control approach differs from quantitative systems pharmacology (QSP) due to the optimization and its quantitative therapeutic goal. The limitations of numerical optimization algorithms mean that optimal control systems are generally smaller than QSP models, and semi-mechanistic rather than mechanistic. Semi-mechanistic models may include only key populations and interactions, which represent “net effects”, rather than specific mechanisms. Such models are fit-for-purpose, to answer specific questions, and their parameters may be estimated from individual- or aggregate-level data.

A QSP model that includes all currently-known mechanisms in a given setting may have so many parameters that they cannot be estimated from data. It also can take longer to create, and may be too large to perform optimization on. In that case, particular regimens can be selected and tested in silico, and the predictions compared. This approach is sometimes referred to as a standard “guess and check” method. The more therapies and dose levels there are to choose from, the less likely this approach is to identify regimens with the best outcomes. For these reasons, QSP models are often used to address other issues, such as mechanisms of action for efficacy and safety, translation of preclinical results to the clinic, and identification of new biomarkers [2].

**Fig. 1:**
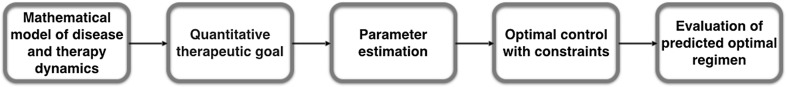
Steps in optimization of drug regimens. Evaluation can be performed by running preclinical (animal) or clinical (human) studies and comparing outcomes to the optimal control predictions.

The general process for applying optimal control is shown in Fig. 1. The first step is to create an appropriate model of the dynamics of the disease and the effects of therapies on the dynamics. The model should be detailed enough to incorporate effects of the particular therapies of interest. Next, the goal of the treatment needs to be quantified. Usually we want to maximize the benefits of the therapies and minimize their side effects. When we combine terms representing these effects, using appropriate signs and weights, we obtain a mathematical expression to be optimized. Once we have determined parameter values to use for the system (see the “Discussion” section for more on this), we can compute the optimal control solution. We should then evaluate the method by comparing outcomes for a predicted optimal regimen with outcomes for standard regimens.

In my husband’s case, the cancer was so advanced that his treatment was considered palliative from the moment of diagnosis. We did not have the time or resources to set up and solve an optimal control problem. Also, there were limited treatment options for his cancer, and most of the chemotherapies used were so toxic, their dosing was limited by the maximum tolerated dose. In such cases, the optimal doses would typically be as high as possible, so I said to Colin’s oncologist, “Throw him under the bus.” Because Colin was so athletic and fit before his diagnosis, we figured he could tolerate the highest doses they would use, which might buy us more time. He suffered side effects, but tolerated the harsh treatments, and got some temporary tumor size reduction. He passed away in February 2012, seven months after his diagnosis [3].

How much difference could optimal control have provided? Probably not much for Colin’s disease stage and limited therapeutic options. But how much difference could optimal control provide more generally? Even without any optimization, standard “guess and check” methods predict that schedule changes could yield significant improvements in combination therapy [4–7]. Clinical data reveal cases in which dose and schedule differences lead to different patient outcomes [4, 8–12]. Below, I present two examples of the types of results predicted using optimal control.

### Example 1

In a model for a hypothetical patient infected with human immunodeficiency virus (HIV), we considered two classes of drugs, protease inhibitors (PIs) and reverse transcriptase inhibitors (RTIs), as well as the development of resistance to them. The mathematical model incorporated an HIV patient’s healthy T cells (immune system cells) and their infection with virus that was either wild-type (not resistant), resistant to just PIs, resistant to just RTIs, or resistant to both. We used optimal control to predict a regimen that would achieve the best possible outcome.

The predicted optimal regimen agreed with the known paradigm for HIV treatment with those drugs, which was “hit early, hit hard”. Initial doses were very high, and were then tapered off. This was compared to a more standard regimen with constant dose levels, but with the same total exposure to the drugs. That is, the optimal regimen was constrained to have the same area under the curve (AUC) as the standard regimen, for each drug.

**Fig. 2:**
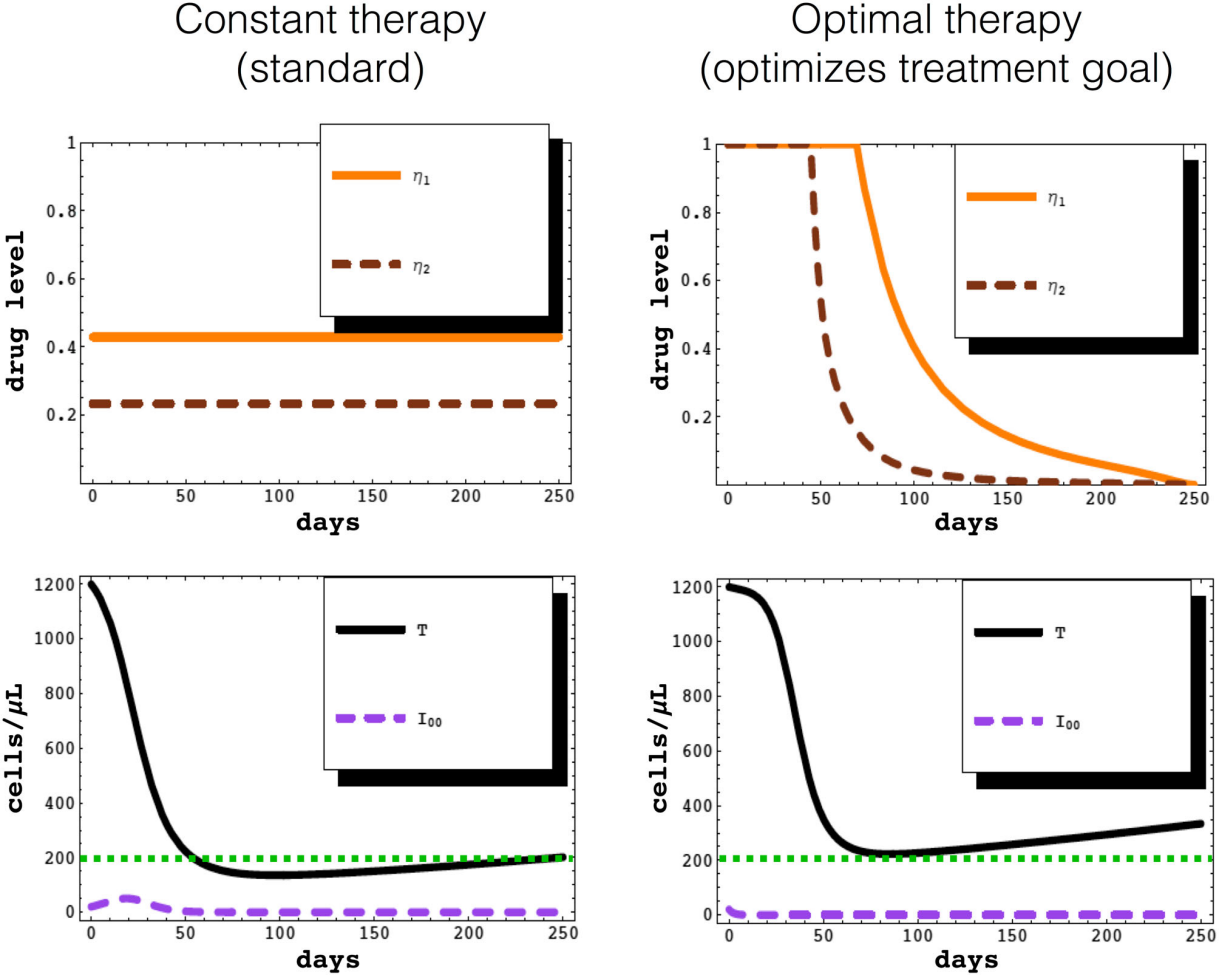
HIV therapy example: How much better can an optimal control regimen be, in comparison to a standard constant-dose regimen? The solid orange curve represents a protease inhibitor ($\eta _1$) and the dashed brown curve represents a reverse transcriptase inhibitor. Each has been scaled so that 0 represents no drug administered and 1 represents a level achieving complete efficacy. The solid black curve represents a healthy T cell population (*T*) and the dashed purple curve represents an infected cell population ($I_{00}$). The dotted green line indicates 200 cells$/\mu L$. Total exposure (area under the curve, AUC) is the same for both regimens, for each drug individually. Both regimens control the infected cell levels, but the optimal regimen gives a better outcome for the patient’s healthy T cell levels. Adapted from [15] (Color figure online)

For the hypothetical patient modeled in this example, the two regimens yield different outcomes (see Fig. 2). With the standard regimen, the patient has CD4+ T cell counts that dip below 200 cells/$\upmu$L, which is the clinical threshold for acquired immunodeficiency syndrome (AIDS). A patient with AIDS is susceptible to life-threatening opportunistic infections. In this state, a salvage therapy could be used to increase the patient’s CD4+ T cell counts, but such therapies generally have harsh side effects [13].

In contrast, the optimal regimen keeps the patient out of AIDS. Additionally, at the end of the therapy, the patient has a CD4+ T cell count that is about 70% higher than if they had been on the standard regimen. See [14, 15] for more details of the model and the application of optimal control. Regimens like the predicted optimal one shown in Fig. 2 (top right) are not typically used in a clinical setting. Although some therapies are administered with variable-dosing programmed into an intravenous pump, most are not. In order to obtain clinically-feasible regimens, constraints are usually imposed, as in Example 2.

### Example 2

Patients with chronic myeloid leukemia (CML) have many treatment options. There are five approved targeted therapies [16–20]. Because these targeted therapies are prescribed for daily use over years, and because resistance to a therapy can arise, one goal that has been attempted is long-term treatment-free remission. There was a suggestion in the literature that combining two targeted therapies might help with that goal [21]. Immunotherapy is an additional treatment option, and has been tested in combination with a targeted BCR-ABL1 inhibitor in treating CML patients [22].

To look for regimens that could help achieve long-term treatment-free remission, we modeled the in-host tumor-immune dynamics of CML. Our fit-for-purpose model was composed of a quiescent leukemic cell population, a proliferating leukemic cell population, and an immune effect (tied to effector T cell levels). We compared various monotherapy and combination therapy regimens for fixed-dose levels. We then used optimal control to predict a regimen that could achieve the best possible outcome. In contrast to the highly-variable drug levels that were allowed in the HIV treatments of Example 1 above, we focused on clinically-feasible regimens. See [23] and [24] for the details of the model and how the clinically-feasible regimens were calculated.

Table 1 compares selected regimens using a measure of how well each one achieved the therapeutic goal in a hypothetical typical patient. This measure (called an objective functional or objective function) incorporates the sizes of the leukemic populations and the amount of each drug administered. Smaller values of the objective functional are better, and the constrained approximation to the optimal regimen is predicted to be about 25% better than the best combination with fixed-dose levels. See [23–26] for more details.

**Table 1:** Chronic myeloid leukemia example: how much better can a constrained optimal control regimen be, in comparison to various standard constant-dose regimens? The drugs represented by *u*_1_ and *u*_2_ are both targeted BCR-ABL1 inhibitors; the drug *u*_3_ represents an immunotherapy adapted from [23]

Regimens (doses in mg)			Value after 5 years
*u*_1_	*u*_2_	*u*_3_	Objective functional
400	0	0	280 × 10^3^
0	140	0	212 × 10^3^
0	0	240	471 × 10^3^
0	70	80	233 × 10^3^
200	70	0	40.7 × 10^3^
200	70	80	37.9 × 10^3^
Constrained approx. to optimal regimen			28.7 × 10^3^

## Background

### History of optimal control

The accomplished mathematician Pontryagin ran an applied math seminar at the Steklov Mathematical Institute in Moscow in the 1950s. One week, two Soviet Air Force engineers showed up with a problem they could not solve: five differential equations with three controls, which modeled the minimal time trajectory of an aircraft [27]. Previous simpler problems had been solved either by ad hoc analytic techniques or by using approximation methods. (In the 2016 movie *Hidden Figures*, mathematician Katherine Johnson of NASA uses Euler’s method to approximate a rocket trajectory changing from elliptic to parabolic, and to calculate the landing position for astronaut John Glenn’s 1962 orbit of the earth [28].)

Pontryagin could not solve the problem the two engineers showed him, and realized he needed a general method to do so. He reportedly worked on the problem during three nights of insomnia and developed the important idea of adjoint functions in Pontryagin’s Maximum Principle [27]. With his former students Gamkrelidze and Boltyanskii, he further developed the theory, and they published it in Russian in various academic journals and a book [29–33]. English translations of the work appeared a short time later. The Soviets did not use the theory for the first two Sputnik satellites, launched in 1957 [27]. It was only after those two launches that they first computed trajectories for re-entry into the earth’s atmosphere. In this way, the theory of optimal control was born out of the need of a high-priority application. The papers of Gamkrelidze [27] and of Pesch and Plail [34] summarize the development of optimal control by Pontryagin’s group.

It turned out the Americans were working on similar problems, and developed related techniques for some special cases. The Research and Development (RAND) Corporation was established in Santa Monica, California after World War II by the US Army Air Forces. It was a nonprofit set up to provide research and analysis to the armed forces [34]. In the early 1950s, a group of mathematicians working at RAND wrote a set of papers [35–38] that held some ideas similar to those the Soviets later developed independently. However, the cases considered by the RAND group were based on the calculus of variations and could not solve the types of problems the optimal control theory developed by the Soviets did, including problems with constraints. The Sputnik and later Soviet satellite launches, and the increased recognition of the importance of mathematical modeling in real-world applications, spurred an increase in scientific and technological initiatives in the US. These included the 1958 National Defense Education Act (NDEA) [39] and the founding of the Defense Advanced Research Projects Agency (DARPA) [40] and the National Aerospace and Science Administration (NASA) [41].

### Applications of optimal control

Today, optimal control is not only used for aerospace—it is also used in automotive, oil and gas, manufacturing, supply chain management, economics, and many other applications with the potential for big savings. This includes systems that model large objects, as well as systems that model a large number of small objects. When even a small percentage improvement would yield substantial benefit, optimal control is a natural tool to apply. Of course, many optimal control solutions provide more than a small percentage improvement.

Some of the first major applications of optimal control outside of engineering were in economics. Merton used optimal control in the 1970s to optimize consumption investment portfolios [42]. The famous Black–Scholes formula for options pricing was derived as the solution to a stochastic optimal control problem [43]. This work became the basis for a boom in options trading in financial markets worldwide. Numerous tests have shown that the prices predicted by the Black–Scholes formula are close to observed prices in many cases [44]. In 1997, Merton and Scholes were awarded the Nobel Prize in Economics for this work [45]. (Black was already deceased at the time of the award.)

Outside of manufacturing applications, optimal control has not yet been adopted widely in the biotechnology/pharmaceutical (biopharma) industry. Phase 2 clinical trials to determine dose levels can cost hundreds of millions of dollars, and their success depends on meeting specified endpoints. Optimal control could be used to predict dose levels and schedules that increase the probability of clinical trial success. However, applications of optimal control to combination regimens have largely been in academic research. For example, see the books by Swan [46], Martin and Teo [47], Lenhart and Workman [48], and Schättler and Ledzewicz [49].

Although the potential for benefit is large, there appear to be two primary reasons for the slow adoption of optimal control: first, the biopharma industry tends to be cautious in adopting new techniques, due to uncertainty around regulatory views; and, second, there have been questions about how well the mathematical models characterize the disease-therapy dynamics. Regarding the second reason, the options pricing models in economics, despite similar concerns, were able to provide significant value, and thus were considered adequate in their characterization of the system that they were modeling. Additionally, mathematical models of pharmacokinetics (drug concentrations over time) and pharmacodynamics (drug effects over time) have continued to improve over the several decades they have been in use. They provide excellent starting points for mathematical models to use for optimal control.

In the past, QSP models were viewed with similar skepticism, and were used by only the most innovative biopharma companies. However, the National Institutes of Health (NIH), in collaboration with leaders in the field, issued a white paper on QSP and its potential applications in drug development in 2011 [2]. In 2014, the US Food and Drug Administration (FDA) used a QSP model to support its request to a sponsor for dose regimen changes [50]. Since these events, more industry resources have been allocated to support the use of QSP models in drug development.

One notable application of control theory in the biopharma industry was the work that formed the basis of the 2016 FDA approval of the “artificial pancreas” for patients with type 1 diabetes [51]. In this system, a glucose monitor samples the patient’s blood sugar levels and provides feedback to the control device (control theory and feedback control are discussed further in the next section). A mathematical model is used to calculate precise amounts of insulin which are administered from the attached insulin pack to help control the glucose levels. Patients still self-inject a large insulin bolus before meals, but the automated and frequent monitoring system that relies on a feedback control model reduces the incidence of dangerous hyperglycemia episodes throughout the day.

The successes of mathematical models for treatment of diabetes, HIV, hepatitis C, and other diseases, have shown that it is possible to create models that capture essential disease-therapy dynamics. Similar to the situation for QSP in the past, optimal control has been applied to biological questions in academia for many years. Efforts have begun in the biopharma industry to apply optimal control to drug regimen optimization. See, for example, the recent work done at Bristol-Myers Squibb [23] (note: this project was ended for reasons unrelated to the optimal control work, before the model predictions could be evaluated with data).

## Theory and an example to illustrate it

In this section, I give some exposition of the theory of optimal control. I also illustrate each step of the procedure by applying it to a previously-published model of therapy for patients with CML [52, 53]. This example was selected because it illustrates the calculations needed for the most common cases.

### What is control theory?

Control theory refers to the ability to change a system in a desired way. A common setting for control theory is a system of ordinary differential equations (a *dynamical system*) that represents *states* we are interested in tracking and changing. We use *controls* to alter one or more states of the system, which will cause a change in the outcome.

For example, the states might be the three positional coordinates of the center of gravity of a rocket, (x, y, z), as governed by gravity and aerodynamics. The controls might be the direction and force produced by a combustion engine. Changing the controls allows us to change the position of the rocket, while it is still being governed by a system that includes the effects of gravity and aerodynamics. If we check the states (the coordinates) at certain times, and use that information to decide how to change the controls, this is called *feedback control*. So if unanticipated wind or debris have affected the position of the rocket, a measurement will reveal the effect, and we can adjust the firing of the engine to take that into account.

A simpler example of feedback control is a water storage tank with a float valve. When the water level rises, the float rises too, and shuts off an input valve. When the water level drops, the float drops too, and the input valve is opened, allowing more water to flow in. In this case, the feedback control is automatic. The water level is the state of interest, and the input water is the control.

Likewise, thermostats can be made with metals or gasses that expand with heat. The thermometer is configured so that when the ambient temperature is high enough, the expansion causes the electrical circuit to open, which causes the furnace to stop running. When the ambient temperature drops enough, the material contracts, the circuit is completed again, and the furnace is triggered by the electrical signal to turn on. The temperature is the state, and the heat generated by the furnace is the control.

The artificial pancreas described above is another example of feedback control. The glucose level is the state, and the insulin input is the control.

### What is optimal control?

Optimal control tries to find the controls (which may vary over time) that get the system as close as possible to a desired outcome. The desired outcome is quantified by an objective functional that is maximized or minimized. The word *functional* simply means the objective is a function of one or more functions. While the objective is still a function, the term functional is more precise, just as the term square is more precise (when applicable) than rectangle. Optimal control uses the same type of state and control functions that are used in control theory, but we add the objective functional and optimize it while the system behaves according to specified equations.

### Semi-mechanistic dynamical systems models of diseases

The dynamical systems of interest in drug development are those that represent states related to diseases. For example, in the case of a cancer of the blood, the concentration of cancerous cells in a patient’s peripheral blood could be a state we are interested in. We can incorporate anti-cancer treatments as controls in the system. In the dynamics of cancer and therapy, there are host immune system cells that play important roles, and they would be included as states as well. The idea of a “minimal model” that captures the key characteristics of the state and control dynamics leads us to “semi-mechanistic models” [49, p. 38].

The model in [53] is semi-mechanistic and includes cancer cells, *C*(*t*), and two types of immune system cells: naive T cells, $T_n(t)$, and effector T cells, $T_e(t)$. Each of the cell types is dependent on time *t*, and time-dependent drug levels (controls) are denoted by $u_1(t)$ and $u_2(t)$. The relationships between the cell concentrations and the controls are represented in the differential equations shown here:

1 $$ \frac{dT_n}{dt} = s_n - u_2 d_n T_n - k_n T_n \left( \frac{C}{C + \eta }\right) $$

2 $$ \frac{dT_e}{dt} = \alpha _n k_n T_n \left( \frac{C}{C + \eta }\right) + \alpha _e T_e \left( \frac{C}{C + \eta }\right) - u_2 d_e T_e - \gamma _e C T_e $$

3 $$ \frac{dC}{dt} = (1 - u_1) r_c C \ln \left( \frac{C_{\max }}{C}\right) - u_2 d_c C - \gamma _c C T_e $$

where $T_n(0)$, $T_e(0)$, and *C*(0) are known. The parameters $s_n$, $d_n$, $k_n$, $\eta$, $\alpha _n$, $\alpha _e$, $d_e$, $\gamma _e$, $r_c$, $C_{\max }$, $d_c$, $\gamma _c$ are all assumed to be non-negative constants. More information about the system and the parameters is given in Moore and Li [52] and Nanda et al. [53].

Because the states of interest are $T_n$, $T_e$, and *C*, Eqs. (1)–(3) are called the *state equations*. In modeling a physical system, it is common that the known information is about local interactions. For example, the last term of Eq. (3) is comprised of three factors multiplied by each other: the constant parameter $\gamma _c$; the concentration of cancer cells, *C*; and the concentration of effector T cells, $T_e$. We used this *mass action* form because we were modeling cell contact-dependent killing of cancer cells by effector T cells. The parameter $\gamma _c$ takes into account both the rate at which effector T cells and cancer cells have encounters, and the proportion of those encounters that lead to the loss of the cancer cell.

By modeling rates of local interactions and events, we get expressions for the rates of change, such as those represented in Eqs. (1)–(3). Solving the system of differential equations means solving for the cell populations whose rates of change we modeled. So we start with differential equations composed of local, instantaneous information, and then solve to obtain functions that describe the cell population levels over time. In the examples detailed in this work, fixed values are used for the parameters. The selected values are intended to represent a typical patient. Methods for handling differences and uncertainty in parameter values are included in the “Discussion” section.

### Objective functionals

In addition to a mathematical model for the system we wish to control, we also need a mathematical model for the treatment goal or objective. For a disease such as cancer, it could be important to minimize the cancer cell levels during and at the end of the treatment period. For the immune cells in the model, we may wish to keep their levels from being too low at the end of the treatment period. And therapies generally have a risk of side effects, so we don’t want to use more than necessary during the treatment period. To put all of these goals together, we decide on a sign (positive or negative) and a relative weight for each goal and add the quantities we wish to minimize.

For example, for the system above, our treatment goal might be expressed as minimizing *J*, where

4 $$ J(u_1, u_2) = \int _0^{t_f} \left[ C(t)+ \frac{B_1}{2} u^2_1(t) + \frac{B_2}{2} u^2_2(t) \right] dt + B_3 C(t_f) - B_4 T_n(t_f) ,$$

where each $B_i$, $i = 1, 2, 3, 4$, is a positive relative constant weight, and $t_f$ is the end time of the treatment period. We wish to minimize terms in the objective *J*; since the naive T cells appear in a negative term, minimizing this term maximizes the naive T cell concentration at the end of the treatment period. The controls $u_1$ and $u_2$ appear inside the integral as quadratic or squared terms for convenience. This choice is less common these days, and is examined further in the “Discussion” section.

The sizes of the relative weights reflect the importance of the various terms in the therapeutic goal. Generally, we rely on disease knowledge to decide on values for the weights. Decision analysis is a formal approach to quantifying this knowledge [54]. Alternatively, ranges of values can be sampled for the weights, yielding qualitative information about patterns of optimal regimens. Marler and Arora [55] examine ways to decide on relative weights in the objective functional in this and more general settings.

Because the treatment goal depends on $u_1$ and $u_2$, which are functions of time, *J* is called a functional (recall this means it is a function of one or more functions). *J* also depends on *C* during the treatment period, but *C* is determined by the dynamical system given by the state equations (1)–(3). The functions $u_1$ and $u_2$ are the only quantities we can control, so they are the inputs, and we consider *J* to be a function of them, $J(u_1, u_2)$. Once we have determined an expression for the objective functional *J*, we want to optimize it. In this example, we optimize by minimizing *J*. To do that, we will take derivatives and set them equal to zero. However, to maintain the underlying dynamical system at the same time, we need the theory of optimal control.

### Optimal control

The key idea behind optimal control is the way the dynamical system and the objective functional are tied together through the *adjoint functions*. To organize the necessary calculations for optimal control, we first form the Hamiltonian *H* (so-called because of its similarity to the Hamiltonian in classical mechanics; cf. [56]). The Hamiltonian is a functional that provides a convenient way to record and combine information about the objective functional and the underlying system dynamics. It combines the right-hand sides of the state equations with the derivative of the objective functional, using the adjoint functions to multiply the state equation components. The theory of optimal control that Pontryagin developed specifies what to do to *H* to obtain controls $u_i$ that optimize the objective functional. In particular, certain derivatives of *H* define the adjoint functions through differential equations (the *adjoint equations*). See the book of Lenhart and Workman [48] for a readable beginner’s introduction to these ideas in optimal control applied to a general setting.

For concreteness, we show the Hamiltonian for the system and objective functional considered above, which demonstrates how to handle common forms:

5 $$\begin{aligned} H= & {} \,C + \frac{B_1}{2}u^2_1 + \frac{B_2}{2} u^2_2 + \lambda _1 \left( s_n - u_2 d_n T_n - k_n T_n \left( \frac{C}{C + \eta } \right) \right)  \\&+\, \lambda _2 \left( \alpha _n k_n T_n \left( \frac{C}{C + \eta } \right) + \alpha _e T_e \left( \frac{C}{C + \eta } \right) - u_2 d_e T_e - \gamma _e C T_e \right)  \\&+ \,\lambda _3 \left( (1 - u_1) r_c C \ln \left( \frac{C_{\max }}{C} \right) - u_2 d_c C - \gamma _c C T_e \right) \, . \end{aligned}$$

The factors $\lambda _1$, $\lambda _2$, and $\lambda _3$ are the adjoint functions, and they are functions of time *t*, as are the state functions $T_n$, $T_e$, and *C* and the control functions $u_1$ and $u_2$. The adjoint functions are used to bring the underlying system dynamics into the optimization (note that they are multiplied by the right-hand sides of the state equations (1)–(3)). The first three terms of *H* are the terms that are inside the integral in *J*. The other two terms of *J* that are not inside the integral contribute the additional *transversality conditions* that accompany the adjoint equations. Namely, they give the conditions $\lambda _1(t_f) = -B_4$, $\lambda _2 (t_f) = 0$, and $\lambda _3(t_f) = B_3$. When the adjoint equations are combined with these final-time conditions, they specify the adjoint functions $\lambda _i$ uniquely, just as the state equations and their initial conditions specify the state functions uniquely.

Thanks to the way *H* is defined, the adjoint equations can be expressed in terms of *H*:

6 $$ \frac{d \lambda _1}{dt} = -\frac{\partial H}{\partial T_n} \, , \, \, \frac{d \lambda _2}{dt} = -\frac{\partial H}{\partial T_e} \, , \, \, \frac{d \lambda _3}{dt} = -\frac{\partial H}{\partial C} , $$

where $\frac{\partial H}{\partial V}$ denotes the partial derivative of *H* with respect to the variable *V*, for $V = T_n$, $T_e$, or *C*. (As a reminder, the partial derivative of *H* with respect to *V* is calculated by treating every parameter or variable *except* *V* as a constant, and then taking the derivative of *H* as usual with respect to *V*.) For the leukemia example considered here, the reader can check that computing the partial derivatives specified in (6) gives the following adjoint equations:

7 $$ \frac{d \lambda _1}{dt} = \lambda _1 \left( u_2 d_n + k_n \frac{C}{C + \eta } \right) - \lambda _2 \alpha _n k_n \frac{C}{C + \eta } , $$

8 $$ \frac{d \lambda _2}{dt} = \lambda _3 \gamma _c C - \lambda _2 \left( \alpha _e \frac{C}{C + \eta } - u_2 d_e - \gamma _e C \right) , $$

9 $$\begin{aligned} \frac{d \lambda _3}{dt}= & {} \lambda _1 k_n T_n \frac{\eta }{(C + \eta )^2} - 1  \\&-\, \lambda _2 \left( \alpha _n k_n T_n \frac{\eta }{(C + \eta )^2} + \alpha _e T_e \frac{\eta }{(C + \eta )^2} - \gamma _e T_e \right)  \\&- \,\lambda _3 \left( (1 - u_1) r_c \left( \ln \left( \frac{C_{\max }}{C} \right) - 1 \right) - u_2 d_c - \gamma _c T_e \right) . \end{aligned}$$

Now we have defined all the needed pieces and can state the problem fully. The problem is to find the drug levels $u_1(t)$ and $u_2(t)$ (which may vary over time) that minimize the objective functional *J* for the disease-therapy system governed by Eqs. (1)–(3). To achieve this, we take the partial derivatives of the Hamiltonian *H* with respect to $u_1$ and $u_2$ and set them equal to zero. That is, we compute the optimal regimens $u_1$ and $u_2$ for this system by setting $\frac{\partial H}{du_1}$ and $\frac{\partial H}{du_2}$ equal to zero and solving for $u_1$ and $u_2$. For our example leukemia model, these equations give:

10 $$ \frac{\partial H}{\partial u_1} = B_1 u_1 - \lambda _3 r_c C \ln \left( \frac{C_{\max }}{C } \right) = 0 , $$

11 $$ \frac{\partial H}{\partial u_2} = B_2 u_2 - \lambda _1 d_n T_n - \lambda _2 d_e T_e - \lambda _3 d_c C = 0 . $$

Solving Eqs. (10) and (11) for $u_1$ and $u_2$ give:

12 $$ u_1 = \frac{\lambda _3 r_c C \ln (\frac{C_{\max }}{C })}{B_1} , $$

13 $$ u_2 = \frac{\lambda _1 d_n T_n + \lambda _2 d_e T_e + \lambda _3 d_c C}{B_2} . $$

We combine these solutions with any lower or upper bounds on $u_1$ and $u_2$ to obtain piecewise-defined functions for $u_1$ and $u_2$ in terms of the state and adjoint functions. Although it may look like we have explicit formulas for the controls (the $u_1$ and $u_2$ functions) and are done, in fact we need to know the state and adjoint functions over time. The state functions are given by Eqs. (1)–(3) and their initial values (at time $t = 0$). However, Eqs. (1)–(3) depend on the controls. The adjoint functions are given by Eqs. (7)–(9) and their final values (at time $t = t_f$), and Eqs. (7)–(9) depend on the state functions. With all of the interdependencies between the control, state, and adjoint functions, the optimal control solutions generally have to be computed using numerical approximation methods.

**Fig. 3:**
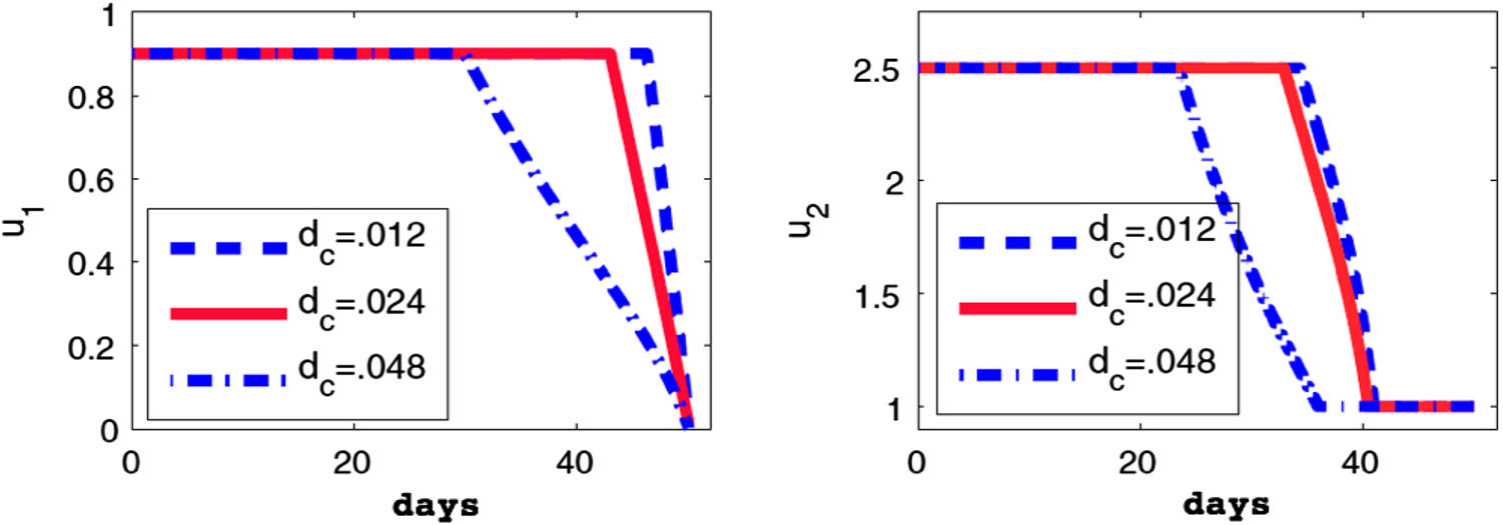
Numerical solutions for $u_1$ and $u_2$ for various values of parameter $d_c$. Three different choices for the sensitive parameter $d_c$ give different optimal regimens for the therapies $u_1$ and $u_2$ for a hypothetical patient. From [53]

One iterative approximation method starts with guesses for $u_1$ and $u_2$. For example, we might guess that both control functions are constant, with $u_1=0.9$ and $u_2=2.5$. We can then solve Eqs. (1)–(3) for the state functions, which allows us to solve Eqs. (7)–(9) for the adjoint functions. These state and adjoint functions can be used in Eqs. (12) and (13) to calculate the control functions. These updated controls can be used to start the process all over again. Once the iterative process results in no more changes in the controls (up to a specified tolerance), then we have found the optimal controls. We can plot these numerical solutions for $u_1$ and $u_2$ (see Fig. 3), as well as the cell levels $T_n$, $T_e$, and *C* over the treatment period [53].

## Discussion

A number of software packages are available for solving optimal control problems. Some of the better-known packages include PROPT (Tomlab, Vasteras, Sweden), DIDO (Elissar Global, Carmel, CA, USA), and GPOPS II (RP Optimization Research, Gainesville, FL, USA), all three of which require Matlab (MathWorks, Natick, MA, USA); there is also the open-source program PSOPT written in C++ (Victor M. Becerra, University of Portsmouth, UK). These packages all use approximation methods based on pseudospectral collocation, and can solve complex optimal control problems. For smaller problems like the ones described in this paper, it is also possible to write a forward-backward iterative loop as described at the end of the previous section. I wrote the code for the problem in Gu and Moore [15] as a loop in Mathematica (Wolfram Research, Champaign, IL, USA). For the problem in Moore et al. [23], we used Tomlab and Matlab. For modelers beginning work in optimal control, I recommend this latter approach. However, for the types of examples discussed in this article (up to five states, up to three controls) any of these software approaches should work. Because the numerical solving is so challenging, equilibria and stability should be determined before approximating the solutions. This can help avoid local minima for *J*.

The objective functional shown as an example in Eq. (4) includes quadratic terms inside the integral. This form was often chosen in the past because the convexity of the quadratic terms makes certain steps of the optimal control problem easier [49, p. 49]. However, methods for handling a variety of functional forms are commonly used now. Thus if toxicity risk for a drug *u*(*t*) correlates with its exposure, we can use a penalty term of the form $\int _0^{t_f} u(t) dt$ (area under the curve, AUC) rather than $\int _0^{t_f} u^2(t) dt$. If toxicity risk of a drug correlates better with the maximum concentration or the time above a threshold concentration *c*, we can incorporate terms of the appropriate form: $\max _{t \in [0, t_f]} u$ or $\int _0^{t_f} f_c(t) dt$ where

14 $$ f_c(t) = \left\{ \begin{array}{ll} 1 &{} {\text{if}} \quad u(t) > c ,\\ 0 &{} {\text{if}} \quad u(t) \le c .\\ \end{array} \right. $$

The regimen optimization examples discussed in this paper all had fixed-time therapy duration; however, optimal control also allows us to include optimization of the duration of the therapy itself by considering an objective functional term with the form $\int _0^{t_f} 1 \, dt$.

There are situations in which pharmacokinetics (PK, the drug concentrations over time) should be incorporated into the mathematical model of the disease and therapy dynamics. In Example 1, this was not done, as the dosing of the drugs was daily and the time period considered was most of a year. For reference, Shudo et al. [57, 2.5–2.6] considered the effect of once-weekly dosing over a period of a few weeks. Models without PK were able to describe the drug effect, with only slight differences from models with PK. For similar reasons, the other examples in this paper also do not include PK. Martin and Teo [47] specifically address the incorporation of PK in optimal control models when it is needed, such as when optimizing the timing of doses.

To integrate optimal control into the development of drug regimens, there are additional considerations that are important for any mathematical modeling we might use. These include tying the model closely to data, handling parameter variability and uncertainty, and evaluating model predictions. I discuss these below, and indicate information unique to optimal control where relevant.

Data-rich settings provide opportunities to capture key disease-therapy dynamics, which are an important first step. Performing sensitivity analysis on the mathematical model can identify parameters that the outcome is largely insensitive to [58, 59]. These “insensitive parameters” can be fixed, and the “sensitive parameters” estimated by fitting the model to data or from the literature.

Nonlinear mixed effects modeling to fit the model to data gives both population information and a set of parameters for each individual. A new population of individuals can be simulated by sampling ranges of parameter values, given specified probability distributions for selection of the parameter values. Computing optimal control regimens for multiple parameter sets (for either previously-studied or simulated groups of individuals) can yield qualitative recommendations for the intended treatment population. *Robust control* is an approach to finding regimens that maintain some level of performance over distributions such as parameter ranges [60]. *Stochastic optimal control* is another method for handling parameter variability, as well as uncertainty [61].

A frequent assumption in pharmacokinetic/pharmacodynamic modeling is that the structure of the equations is the same in animals and humans, and only the parameter values differ. When parameter values in a model are obtained by fitting the model to animal preclinical data, we can use allometric scaling to predict certain corresponding parameter values for a human population (cf. [62]). Alternatively, parameter values can be obtained by directly fitting the model to human clinical data. Optimal control modeling that is tied closely to clinical data includes the work of Swan and Vincent [63], Iliadis and Barbolosi [64], and Zhang et al. [65].

In addition to tying models closely to data, evaluation of a predicted optimal regimen with experimental data is very important. Study outcomes from regimens that are predicted to be optimal should be compared with those from standard regimens. Such evaluation tests whether assumptions in the model, the objective functional, and the parameter estimates, are acceptable and lead to desired outcomes.

## Conclusion

The techniques and examples in this article are intended to support mathematical modelers in the biopharma industry in using optimal control to optimize drug regimens. The papers of Swan and Vincent [63], Kirschner et al. [66], Gu and Moore [15], Nanda et al. [53], and Moore et al. [23] provide additional details for applying optimal control to examples like the ones included in this article. The book of Kamien and Schwartz [67] gives a good exposition of problems like these, as well as more complex problems, such as states with delayed differential equations, state inequalities, integral state equations, and stochastic optimal control.

Optimal control has been applied in numerous industries with great success since its mathematical formulation in the 1950s. Its application to predicting optimal drug regimens or strategies dates back to the 1970s. Recently, it has begun to be used within the biopharma industry to help with the selection of combination regimens.

As with any mathematical modeling approach, the quality of the predictions depends on the quality of the models. With mathematical models that are increasingly able to accurately capture the dynamics of certain disease states, there are expanded opportunities to take advantage of the technique. In planning therapeutic regimens for preclinical or clinical use, we can apply optimal control to these models, to predict optimal regimens based on quantitative therapeutic goals. Current availability of software and expertise makes this feasible for use in the biopharma industry. Thus optimal control is yet another well-established modeling technique we can now leverage more broadly to increase drug development successes, and to give patients the chance for more time with their loved ones.
